# Programmable PCR-like Nonenzymatic DNA Molecular Circuit for Split-Free Autocatalytic Amplification

**DOI:** 10.1021/acs.analchem.5c01612

**Published:** 2025-05-29

**Authors:** Ting Li, Tat San Lau, Junyou Li, Kaiqi Hu, Man Lung Lee, Pin You Chen, Chi Chiu Wang, Hung-Wing Li

**Affiliations:** † Department of Chemistry, 26451 The Chinese University of Hong Kong, Shatin New Territories 999077, Hong Kong SAR, China; ‡ Department of Obstetrics and Gynaecology, The Chinese University of Hong Kong, Shatin New Territories 999077, Hong Kong SAR, China

## Abstract

Nonenzymatic autocatalytic DNA circuits, capable of exponential signal amplification, exhibit superior amplification efficiency compared to traditional single or cascade DNA amplification circuits, which offer only linear or quadratic signal amplification. However, autocatalytic DNA circuits are currently limited by complicated splitting designs, low autocatalytic efficiency, the need for additional probes, and a lack of universal design principles. Herein, we developed a PCR-like split-free autocatalytic amplification (SAA) DNA circuit with a simple design but extraordinary autocatalytic efficiency for effective biosensing. The SAA system could consecutively perform multiple cycles of a three-step process upon initiation, target recognition, replicating, and recycling, which is programmed to mimic the basic three reaction steps of PCR and constantly yield numerous new split-free target replicates to expedite the whole reaction, ultimately producing an exponentially amplified signal. The PCR-like sigmoidal kinetics, the ability to accurately execute the programmed instructions, and the high autocatalytic capability of the SAA system are demonstrated, which achieves the same target replication as the PCR but without the need for enzymes and precise control of temperature. The SAA circuit, characterized by exponential signal amplification, simple design, and minimal components, offers a promising approach for developing highly efficient universal DNA circuits. This enables the analysis of low-abundance biomarkers with minimal signal leakage, holding significant potential for biochemical research and clinical diagnosis.

## Graphical abstract

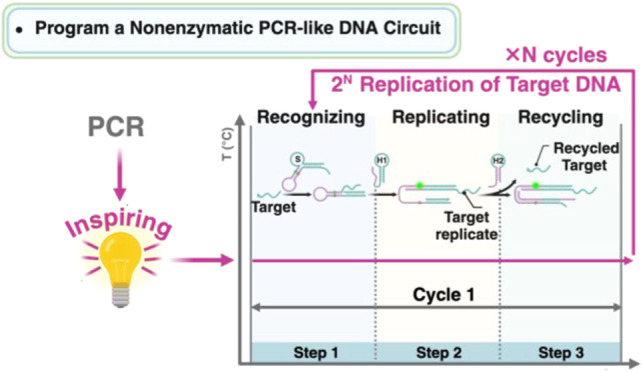

## Introduction

The unprecedented programmability of DNA provides a powerful tool for designing complex and precise DNA-based molecular circuits.^1–4^ DNA circuits can convert DNA strands as inputs to release or synthesize DNA strands as outputs by performing a programmed transformation.^5^ Researchers have developed circuits for many signal-processing functions, including Boolean logic,^6–10^ neural network computation,^11^ and signal amplification.^12–20^

Various isothermal, enzyme-free DNA amplification circuits have been established for tracing diverse biomolecules due to the simplicity and cost-effectiveness of the isothermal, enzyme-free operation. Representative systems, including catalytic hairpin assembly (CHA), hybridization chain reaction (HCR), and advanced cascade circuits like branched HCR, utilize programmable toehold-mediated strand displacement to amplify signals via target sequence recycling or polymer growth.^21–23^ However, most of these circuits predominantly function as signal amplification strategies rather than target amplification strategies. The sensitivity of signal amplification strategies is determined by the turnover number of the process, which limits their performance compared to target amplification techniques, such as polymerase chain reaction (PCR). PCR continuously drives the formation of new target replicates by using polymerase, enabling exponentially amplified detection.^24,25^ As a transformative molecular biology technique, PCR allows the rapid amplification of specific DNA segments through a cyclical process of denaturation, annealing, and extension. While current isothermal, nonenzymatic nucleic acid amplification technologies offer simplicity and signal amplification, they lack the target amplification capability of PCR, which is crucial for achieving enhanced sensitivity.^26^ Thus, designing and programming an enzyme-free DNA circuit that mimics the cycles of PCR for the exponential replication of target DNA sequences and signal amplification is both challenging and significant. Such a circuit would integrate the exponential replication capability of PCR with the simplicity of isothermal enzyme-free operation. This marks a breakthrough in bridging enzymatic and nonenzymatic signal amplification methods and holds substantial promise for molecular diagnostics and point-of-care testing. Most importantly, such an innovative circuit achieves tailored PCR-like replication functionality through programming DNA molecules, which could serve as a valuable model for developing customized DNA circuitry.

Herein, we proposed a nonenzymatic split-free autocatalytic amplification (SAA) DNA circuit, which was programmed to mimic the reaction steps of PCR, enabling the exponential amplification of the target DNA sequence for ultrasensitive detection. Upon the presence of target DNA, the SAA circuit is initiated and undergoes multiple cycles of a three-step process until the reactants are depleted, such as the PCR. At the end of each cycle, a newly exposed target replicate sequence is released, and the original triggered target DNA sequence is also displaced and released, becoming available once more. Consequently, as the SAA progresses, both the newly exposed target replicates and the original target DNA molecules initiate the replication circuit, triggering a chain reaction that leads to the exponential amplification of the original DNA molecule. The SAA system requires only three DNA molecules, each performing distinct tasks in the three steps of the basic cycle. This streamlined and efficient programming ensures that no waste accumulates in the reaction system to interfere with autocatalytic efficiency while also facilitating exploration of the inherent molecular reaction mechanism.

The PCR-like SAA circuit achieves the same functionality as the PCR process but without enzymes and temperature control, enabling one-step, real-time detection of DNA or RNA with picomolar sensitivity and single-base specificity, thereby demonstrating significant potential for disease diagnosis and biological exploration.

## Experimental Section

### Materials

All oligonucleotides were ordered from Sangon Biotech. Co., Ltd. (Shanghai, China) and were purified by high-performance liquid chromatography (HPLC). TE buffer (50 mM) was purchased from Aladdin, China. Magnesium chlorides were purchased from Sigma-Aldrich. All of these chemicals were of analytical grade and used without further purification. Native 12% polyacrylamide gels were purchased from Beyotime, China. GelRed was obtained from Invitrogen. All the solutions prepared were conducted with DEPC-treated water or deionized water (DI water) purified by an ELGA lab water purification system (U.K.) with an electrical resistivity of 18.25 MΩ. The buffer for all experiments was TE (10 mM Tris·HCl, 1 mM EDTA, pH 8.0) with 12.5 mM MgCl_2_ added.

### Fluorescence Assays

Before use, DNA hairpins were heated to 95 °C for 10 min and then cooled to room temperature (25 °C) for 2 h. For the analysis of HCR and SAA, the target DNA was incubated with their respective DNA mixtures (S + H1 for HCR and S + H1 + H2 for SAA, 50 nM S, H1, and H2) for 2 h at 25 °C. The fluorescence spectra were collected from 509 to 645 nm with an excitation wavelength of 488 nm. For sensitive miR-21 detection assay, the miR-21 was added into the mixtures of helper hairpin (Hp) and SAA reactants (12.5 nM Hp and 50 nM S, H1, and H2) at 25 °C for 2 h unless otherwise specified. For kinetically monitoring the fluorescence intensity, the fluorescence intensity at 520 nm (*F*) was measured at appropriate time intervals (every 5 min for 2h and every 15 min for 5h). The fluorescence measurement was performed by a CLARIOstar Plus multimode microplate reader (BMG LABTECH, Germany). The fluorescence change (Δ*F*) is defined as Δ*F* = *F* – *F*_0_, where *F*_0_ is the fluorescence intensity of the system without a corresponding target added.

## Results and Discussion

### Working Principle of SAA Circuit

Three DNA reactants, substrate DNA­(S), hairpin 1­(H1), and hairpin 2­(H2), were thoughtfully programmed to perform recognition, replicating, and recycling of the target DNA sequence, respectively (Scheme 1B). These three steps precisely mimic the functions of the three fundamental steps in the PCR cycle: annealing for primer recognition, extending for the synthesizing new sequence, and denaturing for separating dsDNA (Scheme 1A).

**Scheme 1:**
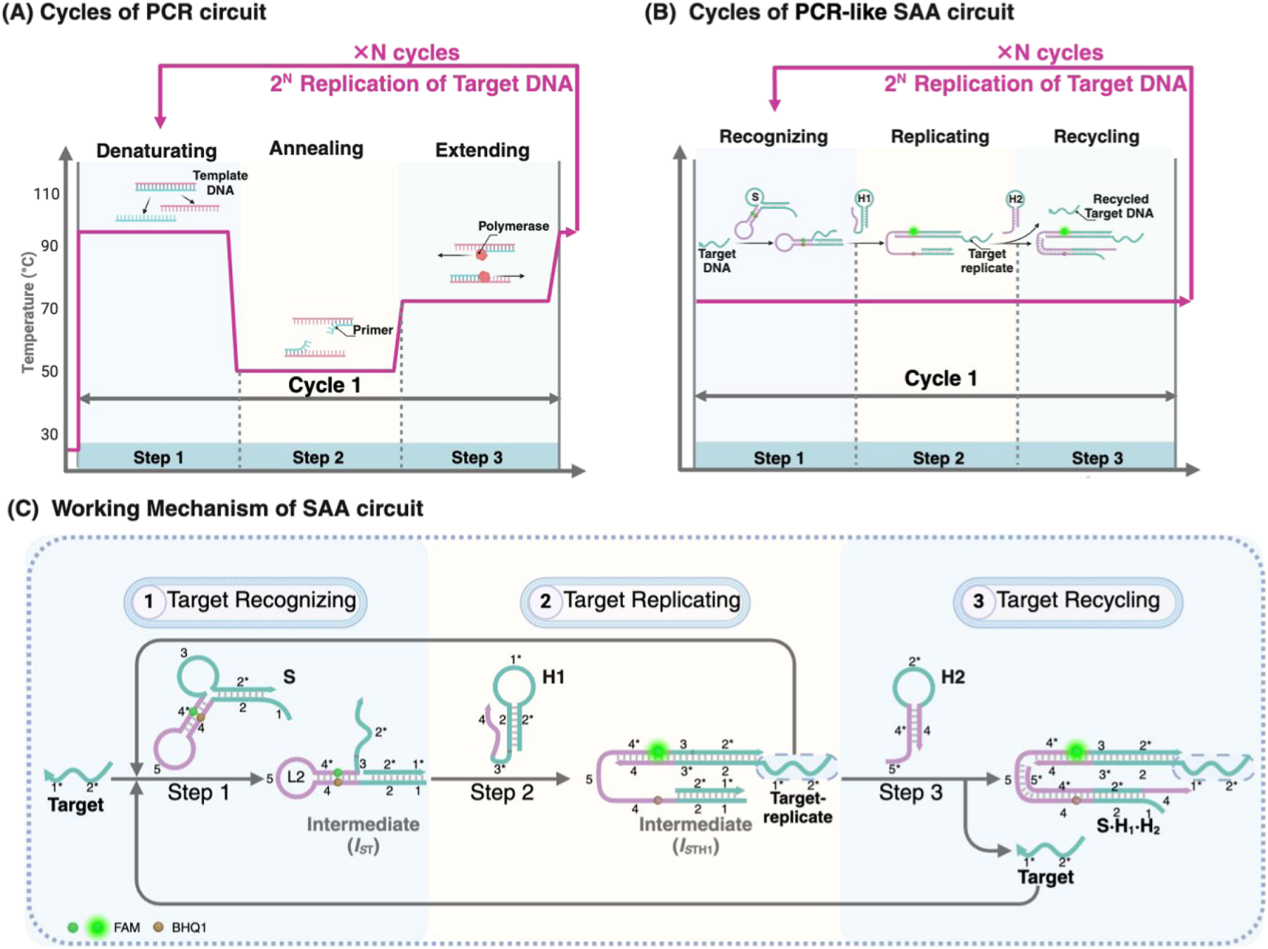
Design and Reaction Mechanism of the Split-Free Autocatalytic Amplification (SAA) Circuit; (A) Schematic Illustration of PCR Reaction Cycles; (B) Schematic Illustration of SAA Reaction Cycles; (C) Detailed Working Mechanism of SAA Circuit

All DNA reactants remained stable, and the circuit could not be initiated in the absence of the target DNA­(T) sequence. However, upon the initiation of T, the circuit then continuously produces the product, a three-stranded assembly (S·H_1_·H_2_), which contains a newly released target replicate (T-repl) sequence, contributing to the system a new T sequence and enabling the exponential amplification of T. As shown in Scheme 1C, a basic cycle contains three steps. In the first recognition step, T specifically recognized the S and opened its bugle loop to form the I_ST_ assembly, whose newly exposed domain (domain 3) could bind to H1 and open H1 by toehold-mediated strand displacement (TMSD), releasing the confined T-repl region­(domain2*-domain1*) of H1 for executing the replicating task of step 2. Simultaneously, the unpaired overhang of H1 (domain 4) could open the sequestered hairpin loop of S via TMSD, causing the separation of fluorophore pairs FAM/BHQ1 on S to recover the FAM fluorescence and generate the I_STH1_ complex with a newly exposed region (domain 4-domain 5). In the final step of the basic cycle, the newly exposed region (domain 4-domain 5) of I_STH1_ could subsequently react with H2 to displace and recycle the originally bound T and form the final product (S·H_1_·H_2_) with a free intact T-repl sequence. Similar to how each newly generated DNA strand in the PCR cycle serves as a template for the next cycle, the newly released T-repl and recycled T in the SAA circuit could also initiate subsequent cycles, ultimately achieving the exponential replication of T and generating amplified turn-on fluorescence. Moreover, the SAA is also an autocatalytic system, and the substantial increase in the signal gain of the SAA system is anticipated to improve the efficiency of detecting trace amounts of analytes.

### Performance Verification of PCR-like SAA Circuit

To verify whether the SAA circuit exhibits PCR-like and autocatalytic behavior, the reaction kinetics were initially monitored through fluorescence experiments. The formation of the product S·H_1_·H_2_ was measured as a function of the initial target DNA concentration (Figure 1A). Notably, sigmoidal product generation curves were observed, which are characteristic of PCR-like, autocatalytic, and self-replicating systems. The reaction rate versus time (Figure 1B) in the case of 100 pM T clearly reveals different phases of the sigmoidal kinetics curve after slow induction followed by rapid exponential amplification, linear growth, and then eventual saturation. It is anticipated that the reaction rate of the initial SAA amplifier was limited by the concentrations of T. As the reaction progresses, the assembled products of S·H_1_·H_2_ containing T-repl contribute plenty of additional new T to the system and thus increase the reaction rate and accelerate the opening of S, generating exponentially increased fluorescence. After that, the reaction rate declined for ultimate exhaustion of all SAA reactants. As with PCR, the time at which the exponential step turns on correlates with analyte concentration (T), shown in Figure 1A. These results verified the successful construction of the PCR-like, autocatalytic system with sigmoidal growth kinetics.

**Figure 1:**
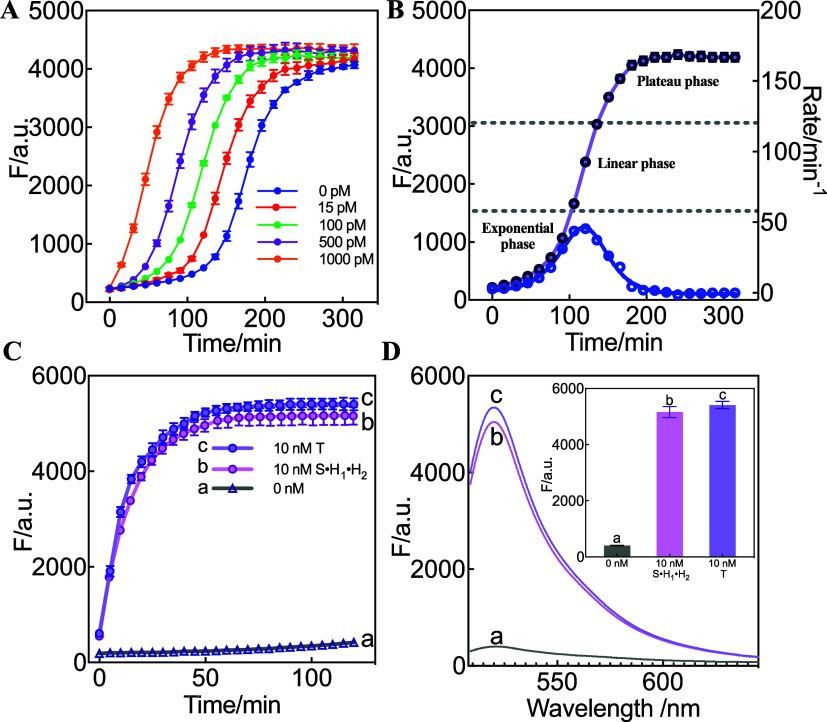
PCR-like performance of the SAA circuit. (A) Time-dependent fluorescence changes of the SAA circuit upon adding the target DNA (T) sequence of varied concentrations. The fluorescence intensity at λ_520nm_ (F) was measured every 15 min. λ_ex_ = 488 nm. (B) Time-dependent fluorescence and rate changes in the case of 100 pM of T. (C) Time-dependent fluorescence changes of the SAA circuit under three conditions: a: without T, b: 10 nM of S·H_1_·H_2_, c: 10 nM of T. The fluorescence intensity at λ_520 nm_ was measured every 5 min. (D) Corresponding fluorescence spectra at 120 min are shown in panel (C). a: without T, b: 10 nM of S·H_1_·H_2_, c: 10 nM of T. Inset: the corresponding fluorescence changes (at λ_520nm_). Error bars represent the mean ± SD from *N* = 3 experiments.

In addition, the SAA circuit, as a PCR-like enzyme-free amplification circuit, demonstrates the feasibility of simulating enzymatic circuits through rationally programmed nonenzymatic circuits. As shown in Table 1, the SAA circuit provides a good example of connecting programmable nonenzymatic and enzymatic circuits. The PCR-like SAA circuit ingeniously achieves the same function of target DNA replication as the PCR but with significant advantages, reacting under isothermal, enzyme-free conditions, which eliminates the need for enzyme and precise temperature controlling. This innovative approach not only simplifies the procedure but also offers valuable insights and inspiration for achieving specific functions through programming of DNA circuits.

**Table 1:** Comparison of PCR and PCR-like SAA Circuit

amplification methods	basic steps	control parameters
PCR-like SAA circuit	recognizing	enzyme-free, isothermal conditions
	replicating	
	recycling	
PCR	annealing	60 °C
	extending	65 °C, polymerases
	denaturing	95 °C

After confirming the sigmoidal kinetics, the autocatalytic capability of the SAA circuit was evaluated as another significant feature. Autocatalytic systems are known for their strong signal amplification, but nonenzymatic autocatalytic systems often suffer from low efficiency due to the split design of target replicates, which can impair recognition or amplification.^27–29^ Examples include self-replicating DNA systems based on HCR^30^ and autocatalytic assembly systems using three-armed catalytic hairpin assemblies.^31^ In contrast, our proposed SAA circuit introduces a split-free design, which means that the T-repl in the S·H_1_·H_2_ product is an intact sequence identical to the original target sequence. The innovative split-free design is anticipated to exhibit superior autocatalytic performance. Nonfluorescent S·H_1_·H_2_ assemblies were prepared by replacing fluorescently labeled S strands with unlabeled counterparts during annealing of the three SAA components (S, H1, H2) and were used to initiate the SAA system. As shown in Figure 1C, S·H_1_·H_2_ successfully initiated and accelerated the SAA reaction as the T sequence did, demonstrating the autocatalysis characteristics of the SAA circuit. Most importantly, the fluorescence change induced by S·H_1_·H_2_ was close to that induced by an equal amount of T after the reactions reached their plateau (Figure 1C,D). The autocatalytic efficiency $\left(\frac{F_{\mathrm{S\cdot H}1\mathrm{\cdot H}2}-F_{0}}{F_{T}-F_{0}}\right)$ of the SAA system was approximately 95% at 120 min, with minimal signal leakage. This inspiring high autocatalytic efficiency was achieved in the SAA system without any reductant optimization of the split site or the length of the target replicated in other works, which may contribute to the split-free design.

### Underlying Mechanism Exploration of the SAA

After verifying the exponential amplification and autocatalytic capability of the SAA circuit, we further explored the underlying reaction mechanism. As illustrated in Figure 2A, when only S and H1 are used as reactants, the anticipated reaction pattern is a traditional hybridization chain reaction of S and H1, comprising only two-step cycles. This would ultimately yield long dsDNA polymers, T-(S·H1)*_n_*, resulting in an N-fold signal amplification in the presence of T. However, introducing H2 in addition to S and H1 would initiate the whole three-step cycle of the SAA circuit upon T activation, incorporating the target and target replicate recycling feedback pathways. Consequently, the final product will be small three-stranded assemblies, S·H_1_·H_2_, not long dsDNA polymers. At the same time, the activated SAA circuit would lead to a signal amplification that is much higher than that of the HCR system. Based on this hypothesis, native polyacrylamide gel electrophoresis (PAGE) and fluorescence experiments were performed to explore the reaction mechanism.

**Figure 2:**
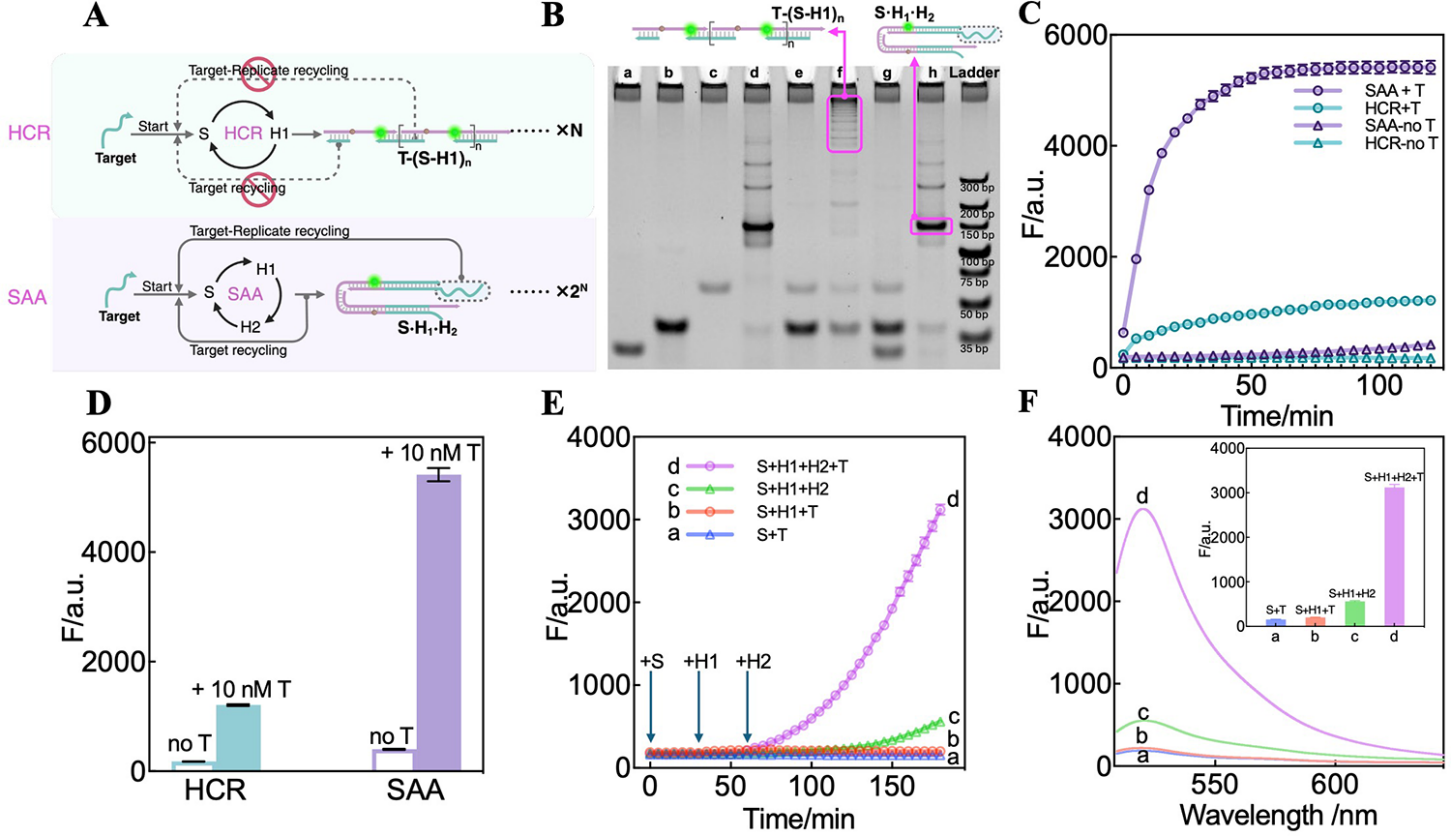
Mechanism exploration of the SAA circuit. (A) Scheme illustration of nonautocatalytic HCR and autocatalytic SAA circuit. (B) Electrophoretic characterizations of the HCR and SAA systems. a: H2, b: H1, c: S, d: Annealed S·H1·H2, e: S + H1, f: S + H1 + T, g: S + H1 + H2, h: S + H1 + H2 + T. (C) Time-dependent fluorescence changes of HCR and SAA in the absence and presence of 10 nM T. The fluorescence intensity at λ_520nm_ was measured every 5 min. (D) Summary of the corresponding fluorescence changes (λ_520nm_) at 120 min shown in panel (C). (E) Time-dependent fluorescence changes after the sequential addition of the reactants. a: 0.5 nM T with S; b: 0.5 nM T, S, and H1; c: S, H1, and H2; d: 0.5 nM T, S. S was added at 0 min for all systems, T was added at 0 min for a,b, and d systems, H1 was added at 30 min for b,c, and d systems, and H2 was added at 60 min for c and d system. The fluorescence intensity at λ_520nm_ was measured every 5 min. (F) Fluorescence spectra of different systems in panel (E) after the addition of all reactants and 120 min of reaction. a: 0.5 nM T with S; b: 0.5 nM T, S, and H1; c: S, H1, and H2; d: 0.5 nM T, S, H1, and H2. Inset: Summary of the fluorescence changes (at λ_520nm_). Error bars represent the mean ± SD from *N* = 3 experiments.

First, PAGE analysis (Figure 2B) revealed that, in the absence of the target DNA, no new bands were observed in either the HCR or SAA systems (lane e and lane g, respectively), indicating that the reactants coexisted stably without unexpected crosstalk. Upon the addition of T, the bands of these reactants turned to be weakened (lane f and h). Besides, new bands of a series of high-molecular-weight products in T-activated HCR (lane f) appeared, suggesting the successfully assembled long linear nanowire products of HCR, T-(S·H1)*_n_*. However, such products in the T-motivated SAA circuit (lane h) were not found. Instead, a distinct band of approximately 150 bp was observed, displaying electrophoretic mobility identical to that of the annealed S·H_1_·H_2_ (lane d), verifying that this newly formed band is the anticipated reaction product of SAA. These results inspirationally substantiate the product difference in the previous mechanism hypothesis. In the absence of H2 for releasing and recycling T and T-repl, S and H1 alternately hybridize after being initiated by T to generate characteristic long linear nanowires as HCR products, T-(S–H1)*_n_* (lane f). However, when H2 is additionally introduced, the third recycling step will be executed, releasing and recycling the T and T-repl confined within the long linear nanowires and breaking down the long linear nanowires into three-stranded S·H_1_·H_2_ complexes for SAA products (lane h).

Fluorescence experiments further validated these findings. Figure 2C displays the time-dependent fluorescence changes with addition of target T (10 nM) to the HCR and SAA systems. In the presence of T, a conventional HCR exhibited a significantly lower fluorescence response than the SAA system. The signal-to-noise ratio­(S/N) of SAA is 13.5, which is almost twice higher than that of HCR (6.8) (Figure 2D). In the comparative experiments of sequential addition of reactants, the results directly revealed the fundamental differences in the amplification pattern and efficiencies between the HCR and SAA. As shown in Figure 2E,F, when H1 was introduced into a mixture of S and T at 30 min to form an activated HCR system (curve b), the fluorescence signal exhibited only a slight increase over time, with a marginal enhancement compared to that of the S and T mixture (curve a). In contrast, upon introducing H2 at 60 min into the system containing T, H1, and S to initiate the SAA system (curve d), the fluorescence signal displayed an initial slow increase followed by a rapid exponential amplification phase. Compared to both the T-activated HCR system (curve b) and the SAA system without T (curve c), the T-activated SAA system (curve d) exhibited significantly higher fluorescence intensity after 120 min reaction, with a distinctive exponentially increasing phase distinguishing it from conventional amplification pattern, validating the difference of amplification capability for HCR and SAA in previous mechanism hypothesis. Thus, the experimental results collectively demonstrated that the SAA circuit has been accurately programmed and operates in accordance with its intended design, ultimately achieving PCR-like, autocatalytic, exponential signal amplification.

### Assessment of the DNA Detection Performance for SAA Circuit

After its exact working mechanism was confirmed, the SAA system was further utilized for the amplified detection of the target DNA sequence under optimal conditions (Figure S2). Figure 3A displays the fluorescence spectra of SAA after reacting with a target T of varied concentrations for 120 min. Clearly, with the increasing concentration of the target DNA, the fluorescence intensity of FAM increased. Also, the change of fluorescence responded to the logarithm of initiator DNA concentrations at a linear relation range from 10 pM–10 nM with a detection limit of 8.9 pM (Figure 3B,C, 3σ-criterion^32,33^ ). Simultaneously, the detection limit of the nonautocatalytic HCR system was acquired as 0.9 nM, which was almost 100-fold higher than that of the autocatalytic SAA system (Figure S3). This enhanced sensitivity was attributed to the autocatalytic feedback pathways of the SAA system, which facilitated the sensitive detection of targets of low content. Moreover, the specificity of the system toward the target DNA was studied to distinguish between target DNA initiators and initiator mutants. Here, single-, double-, and triple mutant targets (SM-T, DM-T, and TM-T) were introduced to evaluate whether the SAA system could discriminate the single-base mutation of the target DNA (Figure 3D). Interestingly, only the target DNA initiated the SAA system with a significantly enhanced readout signal, while these mismatched DNA sequences could not initiate the SAA system, suggesting the high specificity of our SAA system in distinguishing mutant DNA targets.

**Figure 3:**
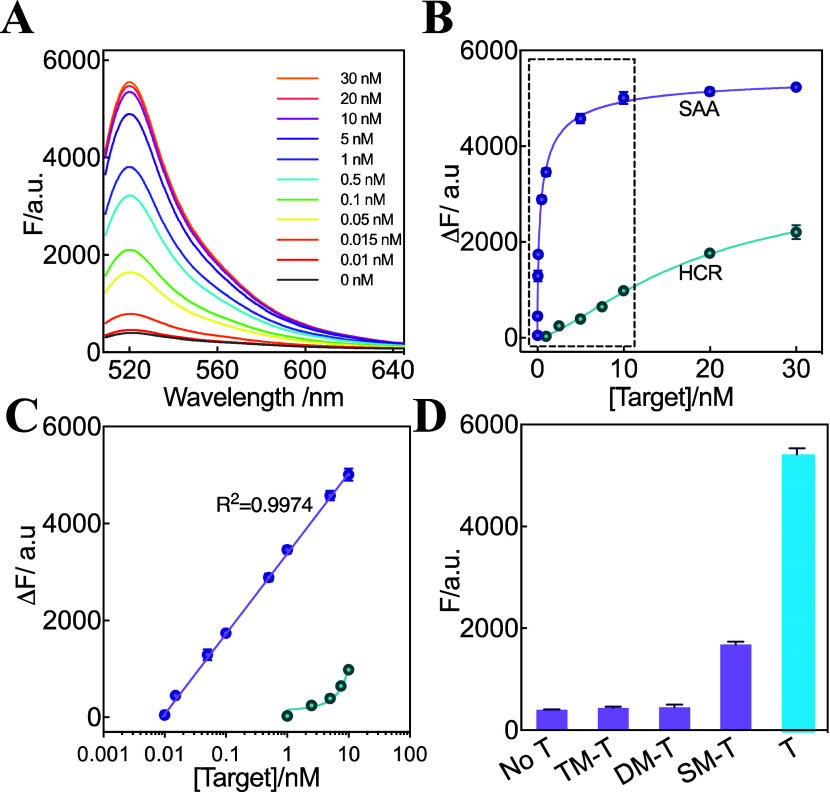
SAA-amplified DNA detection. (A) Fluorescence spectra of the SAA system toward analyzing the target DNA of varied concentrations. (B) Fluorescence change induced by the SAA and HCR circuits upon analyzing different concentrations of target DNA. (C) Calibration curves of the SAA (purple curve) and HCR (green curve) circuits upon analyzing the same target DNA. Linear correlations of SAA can be described as Δ*F* = 1643 lgC_T_ + 3407. (D) Collected fluorescence of the SAA circuit upon analyzing different mutant analytes (10 nM) (λ_520nm_) at 120 min. Error bars represent the mean ± SD from *N* = 3 experiments.

### Assessment of the miRNA-21 Detection Performance for Hp-Transduced SAA Circuit

As a simple and general amplification module, our SAA system can be employed for the determination of other analytes by combination with a facile transduction module. As a proof of concept, the SAA system was extended to analyze the micro RNA-21 (miR-21), which was recognized as a significant oncogene. The overexpressed miR-21 is closely related to the growth, invasion, and metastasis of tumor cells, which makes miR-21 a promising biomarker for early disease diagnosis and therapy.^34–38^ As exhibited in Figure 4A, in the transduction module, the help hairpin (Hp) was utilized to recognize target miR-21. Specifically, miR-21 can open Hp to release T for initiating the SAA-amplified fluorescence response (Figures 4B and S4). Figure 4C displays the fluorescence spectra upon determination of miR-21 with various concentrations. It was observed that the fluorescence increased with the increasing concentration of miR-21 (10 pM–10 nM). A detection limit of 9.2 pM was achieved (Figure 4D), which is equivalent to most of the other approaches for miRNA determination but uses less time and fewer DNA reactants (Table S4). Besides, unlike RT-PCR, the Hp-transduced SAA system directly detects miR-21 without reverse transcription, enabling rapid, isothermal, and enzyme-free workflows ideal for point-of-care applications.

**Figure 4:**
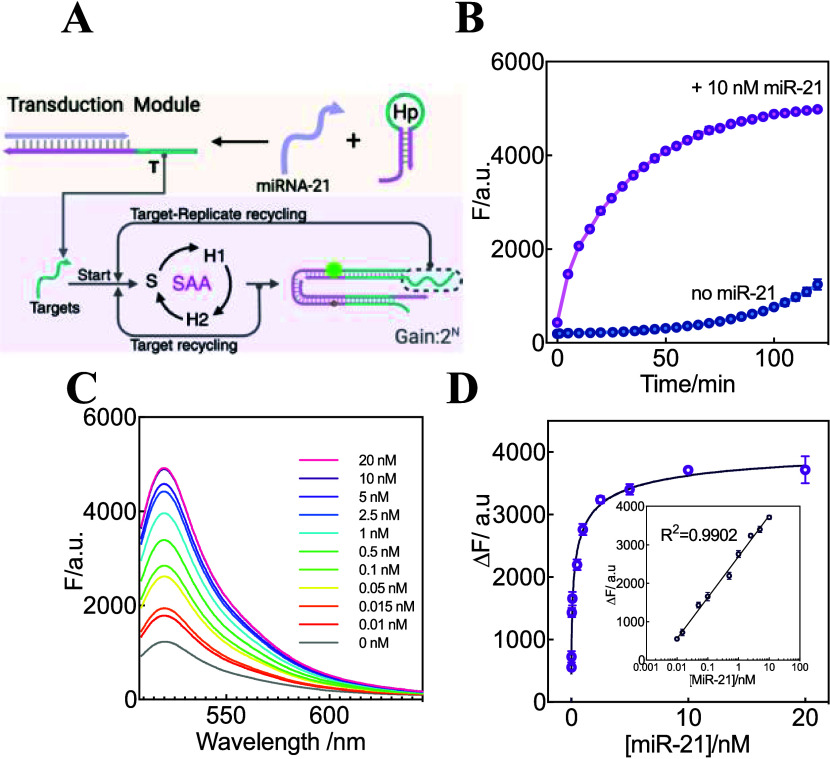
Hp-transduced SAA circuit for miR-21 detection. (A) Scheme illustration of the Hp-transduced SAA circuit for miR-21 detection. (B) Time-dependent fluorescence changes of the Hp-transduced SAA in the absence and presence of miR-21. The fluorescence intensity at λ_520nm_ was measured every 5 min. (C) Fluorescence spectra of the Hp-transduced SAA system toward analyzing miR-21 of varied concentrations. (D) Fluorescence change induced by the Hp-transduced SAA system upon analyzing different concentrations of miR-21. Inset: the corresponding calibration curve. Linear correlations can be described as Δ*F* = 1058 lgC_miR‑21_ + 2696. Error bars represent the mean ± SD from *N* = 3 experiments.

### Analysis of miRNA-21 in Real Samples

To evaluate the practical applicability of the Hp-transduced SAA circuit, its selectivity and specificity toward miR-21 were first tested. Herein, one- and two mutant miR-21, miR-155, let-7a, and miR-221 were selected as mutant miR-21 and potential interfering substances. As shown in Figure 5A, only miR-21 triggered the Hp-transduced SAA system with a significantly enhanced signal, while these mismatched miR-21 and interfering miRNAs failed to activate the system, indicating the good selectivity and specificity of the Hp-transduced SAA system. Next, the performance of the Hp-transduced SAA system was examined in diluted human serum to assess its stability and efficiency in complex biological samples. As shown in Figure 5B, the presence of serum had a minimal impact on the amplification process, indicating that the Hp-transduced SAA system is highly stable and capable of reliable analysis in complex environments. Subsequently, recovery experiments were conducted to detect varying concentrations of miR-21 in 10% diluted human serum. As presented in Table S5, the system achieved high recovery rates ranging from 98.1 to 105.9%, with low relative standard deviations (RSD < 10%). These results demonstrate the accuracy and reproducibility of the Hp-transduced SAA system, highlighting its potential for practical applications in clinical diagnostics and biomarker detection.

**Figure 5:**
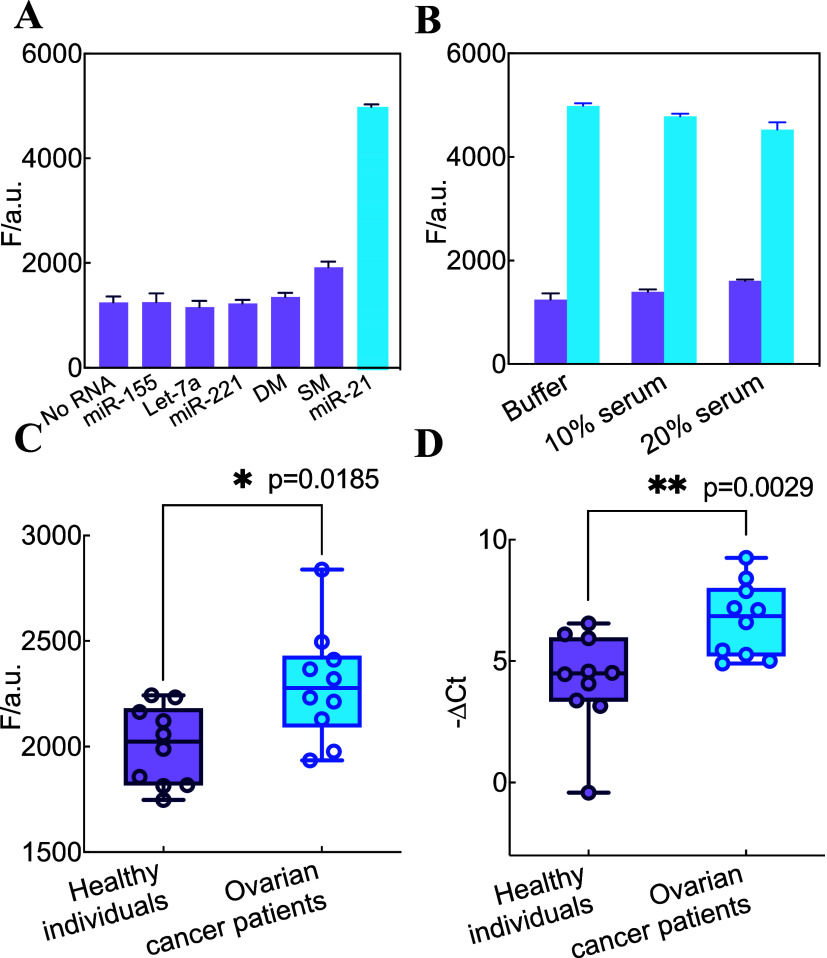
Selectivity, specificity, anti-interference, and clinical applicability of the Hp-transduced SAA circuit. (A) Fluorescence intensity of the SAA amplifier upon analyzing different analytes. SM: one mutant miR-21, DM: two mutant miR-21. (B) Fluorescence intensity of the SAA system upon analyzing miR-21 (10 nM) in the serum (λ_520nm_) at 120 min. (C) Relative expression of miR-21 in serum from ten healthy individuals and ten ovarian cancer patients using Hp-transduced SAA assay. (D) Relative expression of miR-21 in serum from ten healthy individuals and ten ovarian cancer patients using qRT-PCR. miRNA levels were determined from the cycle threshold (Ct) values, and relative miR-21 levels (−ΔCt) were calculated using the equation: −ΔCt = – (Ct value of target miR-21-Ct value of RNU6B). The statistical difference between the two groups was determined using a two-tailed Mann–Whitney test, with *p*-values of >0.05­(ns), ≤ 0.05­(*), ≤ 0.01­(**), ≤ 0.001­(***), ≤ 0.0001­(****) at a 95% confidence interval.

Building on its excellent performance, we further evaluated the clinical applicability of this Hp-transduced SAA system was further evaluated. Previous studies have reported that miR-21 in serum is significantly upregulated in ovarian cancer patients, indicating its potential diagnostic value in ovarian cancer.^39–42^ Thus, we tested the Hp-transduced SAA system in real serum samples from ten healthy individuals and ten ovarian cancer patients. As shown in Figure 5C, the fluorescence responses of ovarian cancer patients are significantly higher than those of healthy individuals, which revealed good agreement with the relative expression of miR-21 in the qRT-PCR result (Figure 5D). These results align well with the reported results, suggesting that the method has the potential to distinguish ovarian cancer patients from healthy individuals. While these preliminary results are encouraging, to conclusively achieve this goal and eliminate random error, expanded clinical trials should be conducted in future studies.

## Conclusions

A programmable PCR-like nonenzymatic autocatalytic DNA circuit with an innovative split-free design was proposed. The intrinsic PCR-like characteristics and autocatalytic capability were explicated through systematic experimental demonstrations. The nonenzymatic SAA circuit not only achieves the same target replication function as PCR without the introduction of enzymes and precision temperature control but also exhibits a high autocatalytic efficiency, reducing reductant optimization steps. The highly sensitive and specific detection of target DNA was attributed to the precise recognition and high autocatalytic efficiency of the SAA circuit. As a universal signal amplification tool, SAA could also detect diverse nucleic acid targets. The Hp-transduced SAA circuit could efficiently detect miR-21 in serum with high sensitivity, selectivity, and anti-interference ability. As a proof of concept, the Hp-transduced SAA system successfully differentiated the ovarian cancer serum samples based on the analysis of miR-21 in real samples.

The unique combination of isothermal enzyme-free operation and exponential autocatalytic amplification characteristics makes the SAA system particularly promising for biomedical analysis and clinical diagnosis through its versatile programmability and field-deployable detection capabilities, such as in vivo molecular monitoring, single-cell analysis via a microfluidic platform, and point-of-care testing in resource-limited settings.

## Supplementary Material



## Notes

Experimental procedures; domain composition and DNA sequences; NUPACK-predicted DNA structures; comparison of different enzyme-free strategies for microRNA detection; recovery test of miR-21; condition optimization; detection performance of HCR (PDF)

## Abbreviations

PCR: polymerase chain reaction
TMSD: toehold-mediated strand displacement
SAA: split-free autocatalytic amplification
T-repl: target replicate
S: substrate DNA
H1: hairpin 1
H2: hairpin 2
T: target DNA
miR-21: micro RNA-21
Hp: help hairpin
