# Supplementary material for: Programmable PCR-like Nonenzymatic DNA Molecular Circuit for Split-Free Autocatalytic Amplification
Source: Anal Chem. 2025 May 29;97(22):11847–55. doi: 10.1021/acs.analchem.5c01612 (PMC12163896; doi:10.1021/acs.analchem.5c01612)
Supplement: Supplementary file 1 [file ac5c01612_si_001.pdf]

Supporting Information

Programmable PCR-like Nonenzymatic DNA Molecular Circuit for Split-Free Autocatalytic Amplification

Ting Li[1], Tat San Lau[2], Junyou Li[1], Kaiqi Hu[1], Man Lung Lee[1], Pin You Chen[1], Chi Chiu Wang[2], and Hung-Wing Li[1]*

[1]Department of Chemistry, The Chinese University of Hong Kong, Sha Tin, New Territories, HONG KONG SAR, China

[2]Department of Obstetrics and Gynaecology, The Chinese University of Hong Kong, Sha Tin, New Territories, HONG KONG SAR, China.

*Author to whom correspondence should be addressed.

E-mail: hungwingli@cuhk.edu.hk

TABLE OF CONTENTS

Experimental Section 2

Table S1. The domain sequences of SAA system 4

Table S2. The domain composition of DNA sequences used in SAA system 4

Table S3. The DNA sequences used in this work 5

Table S4. Comparison of different enzyme-free strategies for microRNA detection 6

Table S5. Recovery test of spiked miR-21 in human serum samples 6

Figure S1. NUPACK-predicted DNA structures of the SAA system.. 7

Figure S2. Optimization of the reaction conditions of the SAA system 8

Figure S3. Detection performance of the conventional HCR system 9

Figure S4. Optimization of the concentration of Hp for miR-21 detection 14

References 15

Experimental Section

Materials

All oligonucleotides were ordered from Sangon Biotech. Co., Ltd. (Shanghai, China) and were purified by HPLC. TE buffer (50 mM) was purchased from Aladdin, China. Magnesium chlorides were purchased from Sigma-Aldrich, USA. All these chemicals were of analytical grade and used without further purification. Native 12% polyacrylamide gels were purchased from Beyotime, China. GelRed was obtained from Invitrogen, USA. All the solutions prepared were conducted with DEPC-treated water or deionized water (DI water) purified by ELGA lab water purification system (UK) with electrical resistivity of 18.25 MΩ. The buffer for all experiments was TE (10 mM Tris · HCl, 1mM EDTA, pH 8.0) with 12.5 mM $MgCl_2$ added.

Fluorescence assays

Before use, DNA hairpins were heated to 95 ˚C for 10 min and then cooled to room temperature (25 ˚C) for 2 h. For the analysis of HCR and SAA, the target DNA was incubated with their respective DNA mixtures (S+H1 for HCR and S+H1+ H2 for SAA, 50 nM of S, H1, and H2) for 2 h at 25 ˚C. The fluorescence spectra were collected from 509 to 645 nm with an excitation wavelength of 488 nm. For sensitive miR-21 detection assay, the miR-21 was added into the mixtures of helper hairpin (Hp) and SAA reactants (12.5 nM of Hp, 50 nM of S, H1, and H2) at 25 ˚C for 2 h unless otherwise specified. For kinetically monitoring the fluorescence intensity, the fluorescence intensity at 520 nm (F) was measured at appropriate time intervals (every 5 min for 2hs, and every 15 min for 5 hs). The fluorescence measurement was performed by CLARIOstar® Plus multi-mode microplate reader (BMG LABTECH, Germany). The fluorescence change (ΔF) is defined as

$\Delta F = F - F_0$, where F_0 is the fluorescence intensity of the system without a corresponding target added.

Native polyacrylamide gel electrophoresis

Gel electrophoresis assay was performed to verify the mechanism of HCR and SAA. 200 nM of hairpin mixtures (S+H1 for HCR and S+H1+ H2 for SAA) were incubated with or without 10 nM of the DNA target in reaction buffer (10 mM Tris · HCl, 1 mM EDTA, 12.5 mM $MgCl_2$, pH 8.0) for 2 h at 25 ˚C. Then, each of these samples was diluted by loading buffer and loaded into 12% native polyacrylamide gel matrix. Electrophoresis was performed at a constant potential of 120 V in 1×TBE buffer (89 mM Tris base, 89 mM Borate, 2 mM EDTA, pH=8.3) for 1.5 h. The gel was imaged by ChemiDoc™ Imaging System (Bio-Rad, USA) under 365 nm UV irradiation after staining in diluted GelRed™ solution for 20 min.

Serum sample collection and storage

Blood samples were collected from patients attending the Department of Obstetrics and Gynaecology, The Chinese University of Hong Kong, Prince of Wales Hospital prior to any treatment after informed consent was obtained. Whole blood was collected into 10 ml red topped clotting tubes (BD Biosciences). Serum was isolated at 3000 g for 10 min at 4 °C. Samples were aliquoted and stored in RNase-free tubes at −80 °C until use. The serum samples were used without pretreatment for miR-21 quantification by SAA.

The qRT-PCR analysis of miR-21 in the serum samples

Total RNA was extracted from serum using Trizol reagent (Invitrogen, US) according to manufacturer's guidelines. The expression of miR-21 and RNU6B was determined by TaqMan MicroRNA Assay (Applied Biosystems) in GeneAmp® 9700 PCR system (Applied Biosystems). Quantitative PCR analysis was carried out using TaqMan® Universal PCR Master Mix and TaqMan® Human MicroRNA Assays (Applied Biosystems). PCR amplification was performed by Lightcyler 480 Instrument (Roche). For each 10 μl PCR reaction, 5 μl of 2× master mix, 1 μl of RT product, 0.5 μl of 20× TaqMan probe for each

miRNA assay and 3.5 μl of milli-Q water was mixed. The reaction mixture was incubated in a 384-well optical plate at 95 ˚C for 10 min, and 60 cycles of 95 ˚C for 15 sec and 60 ˚C for 1 min in Lightcyler 480 Instrument (Roche).

Table S1. Domain sequences of SAA system.

Domain	Sequence	Length (nt)
Domain 1	GGTAAG	6
Domain 1*	CTTACC	6
Domain 2	GGAGAAGTTTGGTGGG	16
Domain 2*	CCCACCAAACTTCTCC	16
Domain 3	TATTAT	6
Domain 3*	ATAATA	6
Domain 4	CGTTGTGATGAGGTTC	16
Domain 4*	GAACCTCATCACAACG	16
Domain 5	GAGTAG	6
Domain 5*	CTACTC	6

Table S2. The domain composition of DNA sequences used in SAA system.

DNA molecules	Domain composition (5'→3')
Substrate (S)	1- 2- 4-5-4*- 3- 2*

Hairpin 1(H1)	2*- 1*- 2- 3*- 4
Hairpin 2(H2)	5*- 4*- 2*- 4
Target DNA(T)	2*- 1*

Table S3. The DNA sequences used in this work.

DNA molecules	Sequence (5'→3')
Substrate (S)	GGTAAGGGAGAAGTTTGGTGGGCGTTGTGAT(BHQ1)GAGGTTCGAGTAGGAACCTCAT(FAM)CACAACGTATTATCCCACCAAACTTCTCC
Hairpin 1(H1)	CCCACCAAACTTCTCCCTTACCGGAGAAGTTTGGTGGGATAATACGTTGTGATGAGGTTC
Hairpin 2(H2)	CTACTCGAACCTCATCACAACGCCCACCAAACTTCTCCCGTTGTGATGAGGTTC
Help hairpin (Hp)	ATCAGACTGATGTTGACCCACCAAACTTCTCCCTTACCTCAACATCAGTCTGATAAGCTA
Target DNA(T)	CCCACCAAACTTCTCCCTTACC
SM-T	CCCACCAAACTTCTCC**G**TTACC
DM-T	CCCACCAAACT**A**CTCC**G**TTACC
TM-T	CCC**TC**CAAACT**A**CTCC**G**TTACC
miR-21	UAGCUUAUCAGACUGAUGUUGA

**SM**	UAGCUUAUCA**C**ACUGAUGUUGA
DM	UAG**G**UUAUCAGACU**U**AUGUUGA
**miR-155**	UUAAUGCUAAUCGUGAUAGGGGU
**Let-7a**	UGAGGUAGUAGGUUGUAUAGUU
**miR-221**	AGCUACAUUGUCUGCUGGGUUUC

The bases of the mutant are highlighted in bold.

Table S4. Comparison of different enzyme-free strategies for miRNA detection.

Amplification systems	Sensitivity/ M	Number of DNA reactants	Sensing duration/h	Ref
Autocatalytic hybridization chain reaction for intracellular imaging of miRNA	1×10^{-13}	6	5	[1]
Concatenated hybridization chain reaction for intracellular imaging	3×10^{-12}	7	2	[2]
Self-Powered and self-Feedback entropy-driven catalyst–DNAzyme circuit	2.1×10^{-11}	3	3	[3]
Autocatalytic, DNAzyme circuit	3×10^{-12}	5	3	[4]
Self-Stacking autocatalyzed assembly circuit	1.7×10^{-12}	4	3	[5]
Autocatalytic hybridization assembly circuit	5×10^{-13}	7	3	[6]
PCR-like split-free autocatalytic amplification DNA circuit	9.2×10^{-12}	4	2	This work

Table S5. Recovery test of spiked miR-21 in human serum samples

Samples	Added amount lg (pM)	Found amount lg (pM)	RSD (%)	Recovered (%)
1	2	2.12 ± 0.02	3.1	105.9
2	3	2.94 ± 0.11	6.8	98.1
3	4	4.17 ± 0.24	5.3	104.1

The detailed design principle of SAA

To construct a PCR-like split-free autocatalytic amplification (SAA) DNA circuit, three DNA molecules containing different domains were precisely designed by NUPACK DNA analysis software. As shown in Figure S1, each DNA reactant showed a relatively stable secondary structure, and S·H1·H2 contained a target replicate overhang as designed.

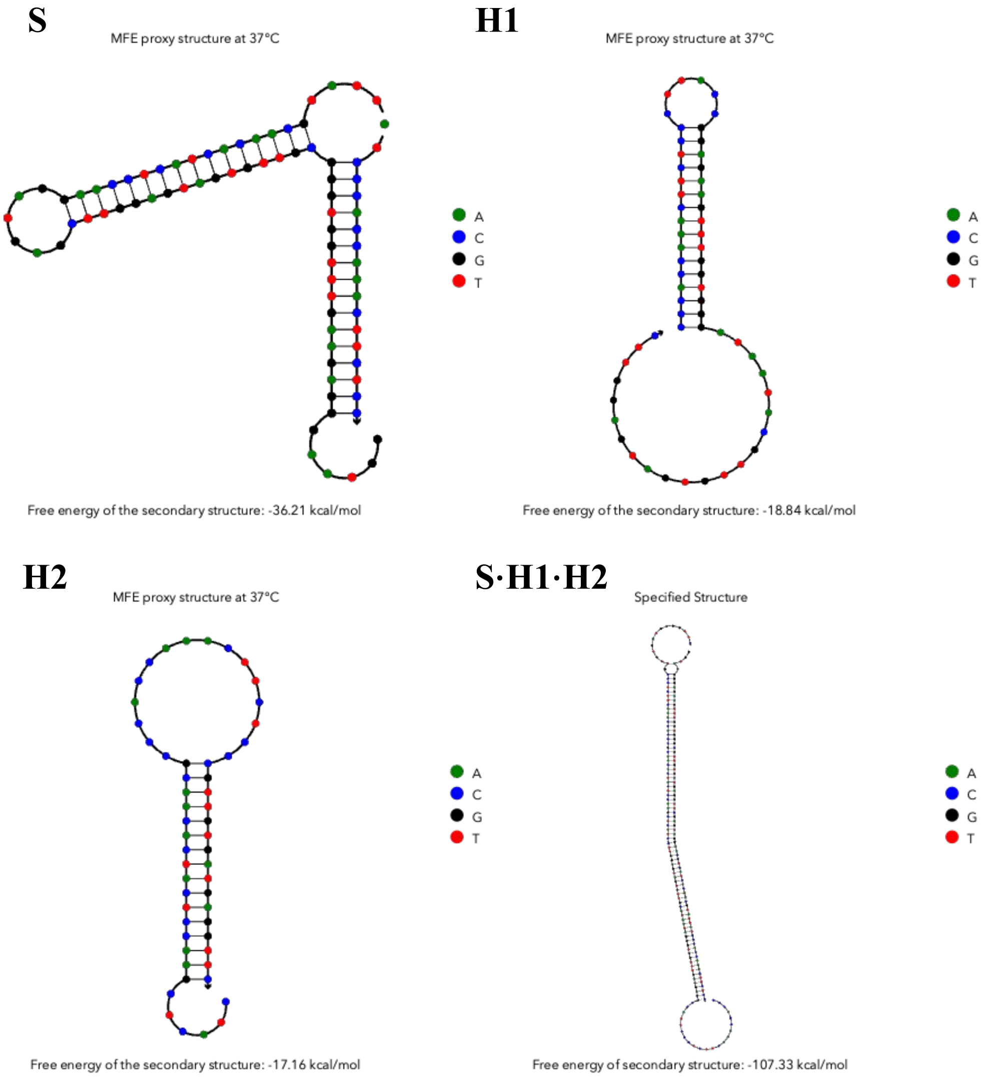

Figure S1. NUPACK-predicted DNA structures of the SAA system. Theoretical simulation of the secondary hairpin structures of S, H1, H2, and the expected product S·H1·H2 at 37 °C by the NUPACK analysis tool.

Optimization of the SAA system

The concentration and stoichiometric ratio of SAA reactants critically influenced both signal amplification efficiency and background leakage. To systematically evaluate these parameters, we investigated the performance of the SAA system under varying concentrations (25–60 nM) and molar ratios of S, H1 and H2, in the presence or absence of trigger DNA (T) at 100 pM and 10 nM concentrations after fixed reaction intervals of 2 h.

As depicted in Figure S2A, increasing reactant concentrations from 25 nM to 60 nM elicited a concomitant rise in both target-triggered fluorescence intensity (100 pM and 10 nM T-activated SAA systems) and nonspecific background signal. Notably, the 50 nM reactant condition achieved an optimal balance between signal gain and low signal leakage. Furthermore, stoichiometric optimization revealed that the S:H1:H2 molar ratio profoundly impacted the reaction. Comparative analysis of multiple ratios (1:1:0.25, 1:1:0.5, 1:1:1, 1:1:1.5, and 1:1.5:1) demonstrated that the equimolar ratio (1:1:1) provided superior signal-to-noise (Figure S2B). This optimal concentration and ratio were applied for all further SAA reactions.

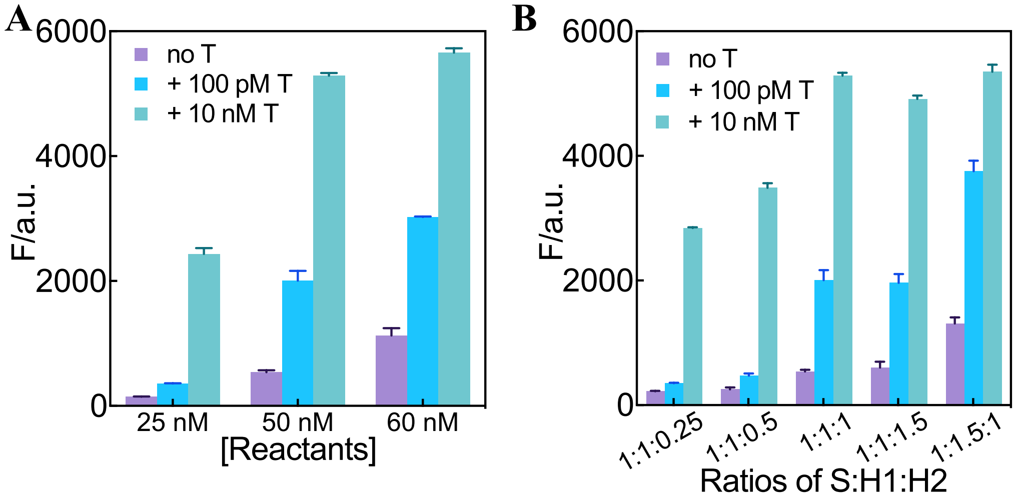

Figure S2. Optimization of the reaction conditions of the SAA system.

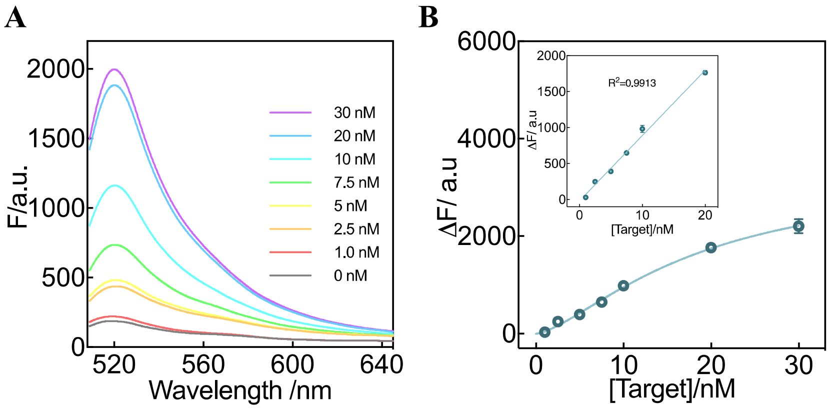

Figure S3. Detection performance of the conventional HCR system (A) Fluorescence spectra of the HCR system toward analyzing different concentrations of DNA target of varied concentrations. (B) Fluorescence change induced by the HCR circuit in the presence of target DNA at different concentrations. Inset: The calibration curve acquired from (A), which could be described as $\Delta F= 90.84\ C_T - 18.76$.

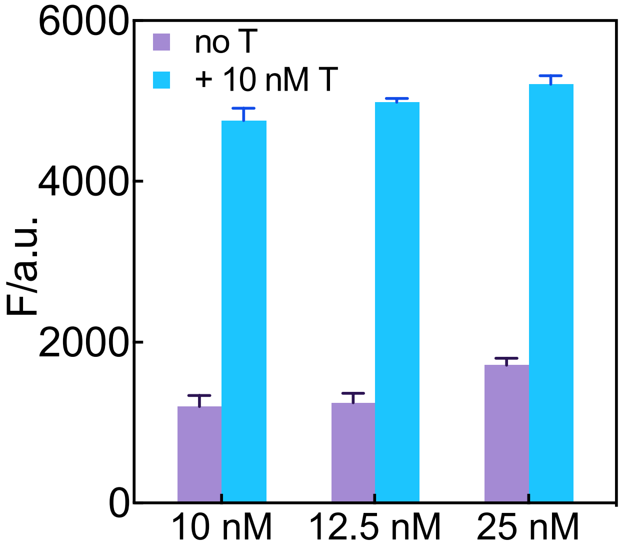

Figure S4. Optimization of the concentration of Hp for miR-21 detection.

References

(1) Wei, J.; Shang, J.; He, S.; Ouyang, Y.; Willner, I.; Wang, F. Construction of a Homogeneous Enzyme-Free Autocatalytic Nucleic Acid Machinery for High-Performance Intracellular Imaging of MicroRNA. *CCS Chem.* **2022**, *4* (11), 3549–3562. https://doi.org/10.31635/ccschem.021.202101545.

(2) Wei, J.; Gong, X.; Wang, Q.; Pan, M.; Liu, X.; Liu, J.; Xia, F.; Wang, F. Construction of an Autonomously Concatenated Hybridization Chain Reaction for Signal Amplification and Intracellular Imaging. *Chem. Sci.* **2018**, *9* (1), 52–61. https://doi.org/10.1039/C7SC03939E.

(3) Xing, C.; Lin, Q.; Gao, X.; Cao, T.; Chen, J.; Liu, J.; Lin, Y.; Wang, J.; Lu, C. Intracellular miRNA Imaging Based on a Self-Powered and Self-Feedback Entropy-Driven Catalyst–DNAzyme Circuit. *ACS Appl. Mater. Interfaces* **2022**, *14* (35), 39866–39872. https://doi.org/10.1021/acsami.2c11923.

(4) Wei, J.; Wang, H.; Wu, Q.; Gong, X.; Ma, K.; Liu, X.; Wang, F. A Smart, Autocatalytic, DNAzyme Biocircuit for in Vivo, Amplified, MicroRNA Imaging. *Angew. Chem. Int. Ed.* **2020**, *59* (15), 5965–5971. https://doi.org/10.1002/anie.201911712.

(5) Li, R.; Zhu, Y.; Gong, X.; Zhang, Y.; Hong, C.; Wan, Y.; Liu, X.; Wang, F. Self-Stacking Autocatalytic Molecular Circuit with Minimal Catalytic DNA Assembly. *J. Am. Chem. Soc.* **2023**, *145* (5), 2999–3007. https://doi.org/10.1021/jacs.2c11504.

(6) Wang, H.; He, Y.; Wei, J.; Wang, H.; Ma, K.; Zhou, Y.; Liu, X.; Zhou, X.; Wang, F. Construction of an Autocatalytic Hybridization Assembly Circuit for Amplified In Vivo MicroRNA Imaging. *Angew. Chem. Int. Ed.* **2022**, *61* (19), e202115489. https://doi.org/10.1002/anie.202115489.
